# Construction of Optimal Frequency Hopping Sequence Set with Low-Hit-Zone

**DOI:** 10.3390/e25071044

**Published:** 2023-07-11

**Authors:** Xinyu Tian, Hongyu Han, Xianhua Niu, Xing Liu

**Affiliations:** 1 College of Computer Science, Sichuan Normal University, Chengdu 610101, China; xytian@stu.sicnu.edu.cn; 2 Visual Computing and Virtual Reality Key Laboratory of Sichuan Province, Sichuan Normal University, Chengdu 610066, China; 3 School of Computer and Software Engineering, Xihua University, Chengdu 610039, China; rurustef1212@gmail.com; 4 College of Electrical Engineering, Sichuan University, Chengdu 610065, China; liuxing4@126.com

**Keywords:** frequency hopping sequence set, low-hit-zone, interleaving techniques, maximum periodic Hamming correlation, frequency-hopping multiple-access

## Abstract

In quasi-synchronous frequency-hopping multiple access (QS-FHMA) systems, low-hit-zone (LHZ) frequency-hopping sequence (FHS) sets have been well-applied to reduce mutual interference (MI). In this paper, we propose three constructions of LHZ FHS sets with new parameters via interleaving techniques. The obtained sequences can be verified that they are optimal with respect to the Peng–Fan–Lee bound.

## 1. Introduction

Frequency-hopping multiple-access (FHMA) is widely used in modern communication systems such as military radar communication systems, Bluetooth communication systems, and more [1,2,3]. Simultaneous transmission of data by multiple users on the same frequency leads to mutual interference (MI), which should be minimized to improve the quality and reliability of the communication. In addition to this, there exists a strong association between the Hamming correlation (HC) of the frequency hopping sequence (FHS) set and the level of the MI. Therefore, it is reasonable to prioritize the construction of FHS sets with a low HC for the significance of the HC in evaluating the performance of frequency hopping sequences (FHSs).

The value of the MI will be maintained at a low level at the zone around the origin between various users, and the low-hit-zone (LHZ) FHS sets will be employed in quasi-synchronous (QS) FHMA systems. Thus, the construction of the optimal LHZ FHS set with respect to the Peng–Fan–Lee bound is preferable to the construction of the optimal FHS set with respect to the Peng–Fan bound [4] in some respects.

Numerous optimal LHZ FHS sets have been found that satisfy the Peng–Fan–Lee bound [5]. Ma and Sun [6] constructed the first class of optimal LHZ FHS sets with respect to the Peng–Fan–Lee bound in 2010. Using the Cartesian, Chung et al. [7] provided a few constructions of the optimal LHZ FHS sets in 2013. By using the interleaving technique, Niu et al. [8,9] obtained various constructions of optimal LHZ FHS sets in 2012 and 2014. Cai et al. [10,11] obtained classes of optimal LHZ FHS sets with optimal partial HC properties in 2014 and 2015. Han et al. [12] and Wang et al. [13] constructed LHZ FHS sets with different parameters in 2016. Using the Cartesian, Zhou et al. [14,15] introduced some constructions of LHZ FHS sets in 2017. Ling et al. [16] obtained a class of optimal LHZ FHS sets in the next year. Niu et al. [17,18] constructed a new class of optimal LHZ FHS sets with large family sizes in 2019. In the same year, the construction by Han et al. [19] of a class of LHZ FHS sets with the optimal partial HC. Niu et al. [20,21] constructed classes of optimal FHS sets in 2020. In 2022, Zhou et al. [22] constructed four classes of LHZ FHS sets with optimal partial HC properties.

In this paper, we propose three constructions of LHZ FHS sets with new parameters by interleaving technique and the LHZ FHS sets are optimal with respect to the Peng–Fan–Lee bound. We make the following arrangement for the remaining portion of this paper. We introduced some notations and the bounds on the FHS set in Section 2. We described how the LHZ FHS set is constructed by using an interleaving technique in Section 3. Finally, we made a few concluding remarks in Section 4.

## 2. Preliminaries

Let $F=\{f_{1},f_{2},\cdots ,f_{c}\}$ be a set with *c* available frequency slots. A sequence $X={\left\{x_{j}\right\}}_{j=0}^{L-1}$ is called a FHS of length *L* over *F* for all $x_{j}$, 0≤j≤L−1. For any two FHSs $X={\left\{x_{j}\right\}}_{j=0}^{L-1}$, $Y={\left\{y_{j}\right\}}_{j=0}^{L-1}$ of length *L* over *F*, their *Hamming correlation function* $H_{XY}\left(\tau \right)$ of sequences *X* and *Y* at delay time τ is defined by

$$\begin{array}{ccc} H_{XY}\left(\tau \right) & = & \sum_{j=0}^{L-1}h\left[x_{j},y_{j+\tau }\right]\;\;\;0\leq \tau \leq L-1, \end{array}$$

where the subscript j+τ of the above equation needs to be modulo *L*, and $h[x_{j},y_{j+\tau }]=1$ if $x_{j}=y_{j+\tau }$, and 0 otherwise.

For a sequence *X*, *the Hamming autocorrelation* of *X* can be defined as

$$\begin{array}{c} H_{a}\left(X\right)=\max_{1\leq \tau \leq L-1}\left\{H_{XX}\left(\tau \right)\right\}. \end{array}$$

For any given FHS set Q, the *maximum Hamming autocorrelation (MHAC)* $H_{a}\left(Q\right)$, the *maximum Hamming crosscorrelation (MHCC)* $H_{c}\left(Q\right)$ and the *maximum Hamming correlation (MHC)* of Q are, respectively, defined as

$$\begin{array}{c} H_{m}\left(Q\right)=\max\left\{H_{a}\left(Q\right),H_{c}\left(Q\right)\right\}, \end{array}$$

$$\begin{array}{c} H_{a}\left(Q\right)=\max_{1\leq \tau \leq L-1}\left\{H_{XX}\left(\tau \right)|X\in Q\right\}, \end{array}$$

$$\begin{array}{c} H_{c}\left(Q\right)=\max_{0\leq \tau \leq L-1}\left\{H_{XY}\left(\tau \right)|X,Y\in Q,X\neq Y\right\}. \end{array}$$

In 2004, Peng and Fan proposed a lower bound for an FHS set as follows.

**Lemma 1**  (Peng-Fan bound [4])**.** *Let Q $\left(L,N,c,H_{m}\left(Q\right)\right)$ be a set, then we have*

$$H_{m}\left(Q\right)\geq \frac{(NL-c)L}{(NL-1)c},$$

*where $\left(L,N,c,H_{m}\left(Q\right)\right)$ denotes a set of N FHSs of length L with size c, with the MHC $H_{m}\left(Q\right)$.*

If each parameter of the FHS set Q substituted into the above equation satisfies the equal sign case, the Q is said to be the optimal FHS set.

For any FHS set Q, if $H_{a}\geq 0$, $H_{c}\geq 0$, the LHZ $Z_{h}$ of Q is defined as

$$\begin{array}{cc} & Z_{h}=\min\left\{Z_{ah},Z_{ch}\right\},\\ & \mathrm{where}\\ & Z_{ah}=\max_{1\leq \tau \leq G}\left\{G|H_{XX}\left(\tau \right)\leq H_{a},\forall X\in Q\right\},\\ & Z_{ch}=\max_{0\leq \tau \leq G}\left\{G|H_{XY}\left(\tau \right)\leq H_{c},\forall X,Y\in Q,X\neq Y\right\}. \end{array}$$

In 2006, Peng, Fan and Lee proposed a lower bound for the LHZ FHS set as follows.

**Lemma 2**  (Peng–Fan–Lee bound [5])**.** *Let $Q(L,N,c,Z_{h},H_{m}\left(Q\right)$ be the LHZ FHS set. Then, for any positive integer Z, $0\leq Z\leq Z_{h}$, we have*

$$H_{m}\left(Q\right)\geq \frac{(NZ+N-c)L}{(NZ+N-1)c},$$

*where $\left(L,N,c,Z_{h},H_{m}\left(Q\right)\right)$ denotes a set of N FHSs of length L with size c, with the MHC $H_{m}\left(Q\right)$ and the LHZ $Z_{h}$.*

If each parameter of the LHZ FHS set Q substituted into the above equation satisfies the equal sign case, the Q is said to be the optimal LHZ FHS set.

## 3. Interleaving Technique of FHSs

Let $A=(a_{0},a_{1},\cdots ,a_{L-1})$ be a $\left(L,c,H_{a}\left(A\right)\right)$ FHS, and $E=(e_{0},e_{1},\cdots ,e_{T-1})$ be a shift sequence of length *T* over a frequency slot set of size *p*, i.e., $e_{i}\in p$, 0≤i<T. A matrix of TL can be obtained through the sequences *A* and *E* in the following way.

(1) $$\begin{array}{cc} \gamma = & \left(\begin{array}{cccc} a_{0+e_{0}} & a_{0+e_{1}} & \ldots & a_{0+e_{T-1}}\\ a_{1+e_{0}} & a_{1+e_{1}} & \ldots & a_{1+e_{T-1}}\\ \vdots & \vdots & \ddots & \vdots \\ a_{L-1+e_{0}} & a_{L-1+e_{1}} & \ldots & a_{L-1+e_{T-1}} \end{array}\right)\\ = & \left(\begin{array}{cccc} b_{0} & b_{1} & \ldots & b_{T-1}\\ b_{T} & b_{T+1} & \ldots & b_{2T-1}\\ \vdots & \vdots & \ddots & \vdots \\ b_{T(L-1)} & b_{T(L-1)+1} & \ldots & b_{TL-1} \end{array}\right). \end{array}$$

Reading each element of the matrix γ by row, we have a sequence $B=(b_{0},b_{1},\cdots ,b_{TL-1})$ of length TL. Let *B* be called the interleaved sequence and *E* is called a shift sequence. The interleaved sequence *B* can be written as

$$B=I\left(L^{e_{0}}\left(A\right),L^{e_{1}}\left(A\right),\cdots ,L^{e_{T-1}}\left(A\right)\right),$$

where *I* is the interleaving operator and *L* is the shift operator.

Let $U=(u_{0},u_{1},\cdots ,u_{T-1})$ be another shift sequence over a frequency slot set of size *p* and $V=I\left(L^{u_{0}}\left(A\right),L^{u_{1}}\left(A\right),\cdots ,L^{u_{T-1}}\left(A\right)\right)$. Considering the shift factor, we can obtain $L^{\tau }\left(V\right)$, where $\tau =T\tau_{1}+\tau_{2}\left(0\leq \tau_{2}<T,0\leq \tau_{1}<L\right)$. By the matrix representation, $L^{\tau }\left(V\right)$ could be written as

(2) $$\left(\begin{array}{cccccc} a_{u_{\tau_{2}}+\tau_{1}} & \ldots & a_{u_{T-1}+\tau_{1}} & a_{u_{0}+\tau_{1}+1} & \ldots & a_{u_{\tau_{2}-1}+\tau_{1}+1}\\ a_{u_{\tau_{2}}+\tau_{1}+1} & \ldots & a_{u_{T-1}+\tau_{1}+1} & a_{u_{0}+\tau_{1}+2} & \ldots & a_{u_{\tau_{2}-1}+\tau_{1}+2}\\ \vdots & \ddots & \vdots & \vdots & \ddots & \vdots \\ a_{u_{\tau_{2}}+\tau_{1}-1} & \ldots & a_{u_{T-1}+\tau_{1}-1} & a_{u_{0}+\tau_{1}} & \ldots & a_{u_{\tau_{2}-1}+\tau_{1}} \end{array}\right).$$

Obviously, $L^{\tau }\left(V\right)$ is just another interleaved sequence. Namely, we have

$$L^{\tau }\left(V\right)=I\left(L^{u_{\tau_{2}}+\tau_{1}}\left(A\right),\cdots ,L^{u_{T-1}+\tau_{1}}\left(A\right),L^{u_{0}+\tau_{1}+1}\left(A\right),\cdots ,L^{u_{\tau_{2}-1}+\tau_{1}+1}\left(A\right)\right).$$

Then, the obtained HC function of the interleaved sequence *B* and *V* at delay time τ can be expressed as the summation of the inner product between the (1) and (2). Then, we have

$$H_{BV}\left(\tau \right)=\sum_{t=0}^{T-\tau_{2}-1}H_{AA}\left(u_{t+\tau_{2}}-e_{t}+\tau_{1}\right)+\sum_{t=T-\tau_{2}}^{T-1}H_{AA}\left(u_{t+\tau_{2}-T}-e_{t}+\tau_{1}+1\right).$$

For any $\tau_{2},0\leq \tau_{2}<T$, let

$$d_{t,\tau_{2}}^{\left(E,U\right)}=\left\{\begin{array}{cc} e_{t}-u_{t+\tau_{2}}, & 0\leq t\leq T-1-\tau_{2}\\ e_{t}-u_{t+\tau_{2}-T}-1, & T-\tau_{2}\leq t\leq T-1 \end{array}\right.,$$

where $d_{t,\tau_{2}}^{\left(E,U\right)}$ needs to be modulo *p*. Then, the HC function of *B* and *V* can be rewritten as

$$H_{BV}\left(\tau \right)=\sum_{t=0}^{T-1}H_{AA}\left(\tau_{1}-d_{t,\tau_{2}}^{\left(E,U\right)}\right).$$

**Lemma 3.**  *According to the above notation, for the sequences B and V, corresponding to the shift sequences E and U, we have*

$$\begin{array}{cc} \; & H_{BV}\left(\tau \right)=TH_{a},\;\mathrm{for}\;\tau_{1}<\min_{0\leq t,\tau_{2}<T}\left\{d_{t,\tau_{2}}^{\left(E,U\right)}\right\}, \end{array}$$

*where $\tau =T\tau_{1}+\tau_{2}\left(0\leq \tau_{2}<T,0\leq \tau_{1}<L\right).$*

Besides, we introduce a class of construction methods by an interleaving technique as follows.

Step 1: Select an $\left(L,N,c,H_{m}\left(A\right)\right)$ FHS set A,
  $$A=\left\{a^{i}=\left(a_{0}^{i},a_{1}^{i},\cdots ,a_{L-1}^{i}\right)|0\leq i<N\right\}.$$
Step 2: For a given *T*, and gcd(L,T)=1, generate a set of shift sequences,
  $$E=\left\{e^{j}=\left(e_{0}^{j},e_{1}^{j},\cdots ,e_{T-1}^{j}\right)|0\leq j<M\right\}.$$
Step 3: Construct the FHS set $B=\{b^{k}|0\leq k<NM\}$, where k=iM+j(0≤j<M,0≤i<N). Then for any 0≤k<NM,
  $$b^{k}=I\left(L^{e_{0}^{j}}\left(a^{i}\right),L^{e_{1}^{j}}\left(a^{i}\right),\cdots ,L^{e_{T-1}^{j}}\left(a^{i}\right)\right).$$

By the above construction, we can deduce the LHZ and MHC of this LHZ FHS set as follows.

**Theorem 1.**  *The sequence set $B=\{b^{k}|0\leq k<NM\}$ generated by the interleaving technique is a $\left(TL,NM,c,Z_{h},TH_{m}\left(A\right)\right)$ LHZ FHS set, where*

$$\begin{array}{cc} \; & Z_{h}+1=\\ \; & \min\left\{\min_{e^{j_{1}}\in E}\left\{\min_{0\leq t,\tau_{2}<T}\left\{Td_{t,\tau_{2}}^{\left(e^{j_{1}},e^{j_{1}}\right)}+\tau_{2}\right\}\right\}\right.,\left.\min_{e^{j_{1}}\neq e^{j_{2}}\in E}\left\{\min_{\left(0\leq t,\tau_{2}<T\right.}\left\{Td_{t,\tau_{2}}^{\left(e^{j_{1}},e^{j_{2}}\right)}+\tau_{2}\right\}\right\}\right\}. \end{array}$$

**Proof of Theorem 1.**  For any two FHSs $b^{k_{1}}$, $b^{k_{2}}$∈B, which correspond to the shift sequences $e^{j_{1}}$ and $e^{j_{2}}$∈E, then the MHC of $H_{m}\left(B\right)$ in the LHZ can be verified as follows.

Case 1: $k_{1}=k_{2}$. Then we have $e^{j_{1}}$ = $e^{j_{2}}$. From Lemma 3, the MHAC of the sequences is $TH_{a}\left(A\right)$, when $0<\tau_{2}\leq \min_{e^{j_{1}}\in E}\left\{\min_{0\leq t,\tau_{2}<T}\left\{Td_{t,\tau_{2}}^{\left(e^{j_{1}},e^{j_{1}}\right)}+\tau_{2}\right\}\right\}$. The case does not need to be concerned when the $\tau_{2}=0$.

Case 2: $k_{1}\neq k_{2}$.

(1) If $a^{i_{1}}\neq a^{i_{2}}$ and $e^{j_{1}}$ = $e^{j_{2}}$, according to the displacement characteristics, the MHCC of the sequences is $TH_{c}\left(A\right)$ for any $\tau_{1}$ and $\tau_{2}$.
(2) If $a^{i_{1}}\neq a^{i_{2}}$, $e^{j_{1}}\neq e^{j_{2}}$ or $a^{i_{1}}=a^{i_{2}}$, $e^{j_{1}}\neq e^{j_{2}}$, the MHCC of the sequences is $TH_{c}\left(A\right)$, for
  $$0\leq \tau <\min_{e^{j_{1}}\neq e^{j_{2}}\in E}\left\{\min_{0\leq t,\tau_{2}<T}\left\{Td_{t,\tau_{2}}^{\left(e^{j_{1}},e^{j_{2}})\right.}+\tau_{2}\right\}\right\}.$$

From the above cases, for any τ, $0\leq \tau \leq Z_{h}$, the MHC $H_{m}\left(B\right)$ of B is given by $H_{m}\left(B\right)=\max\left\{H_{a}\left(B\right),H_{c}\left(B\right)\right\}=\max\left\{TH_{a}\left(A\right),TH_{c}\left(A\right)\right\}=TH_{m}\left(A\right)$, where

$$\begin{array}{cc} & Z_{h}+1=\\ & \min\left\{\min_{e^{j_{1}}\neq e^{j_{2}}\in E}\left\{\min_{0\leq t,\tau_{2}<T}\left\{Td_{t,\tau_{2}}^{\left(e^{j_{1}},e^{j_{2}})\right.}+\tau_{2}\right\}\right\},\min_{e^{j_{1}}\in E}\left\{\min_{0\leq t,\tau_{2}<T}\left\{Td_{t,\tau_{2}}^{\left(e^{j_{1}},e^{j_{1}}\right)}+\tau_{2}\right\}\right\}\right\}. \end{array}$$

 □

## 4. Optimal FHS Set with LHZ

In this section, the optimal LHZ FHS set with the new parameters is constructed based on different shift sequences through the interleaving technique [23,24].

**Construction 1.**  *Step 1: Select an optimal FHS set A$\left(L,N,c,H_{m}\left(A\right)\right)$ that satisfies the Peng–Fan bound, $A=\left\{a^{i}=\left(a_{0}^{i},a_{1}^{i},\ldots ,a_{L-1}^{i}\right)|0\leq i<N\right\}$.*

*Step 2: Let T,u,k be three positive integers, θ is an integer, T>2, uT=L and k=u−1. The shift sequence $E=\left\{e_{i}^{j}|0\leq i<T,0\leq j<k\right\}$. We have*
$$e_{i}^{j}=\left(e_{0}^{j},e_{1}^{j},\ldots ,e_{T-1}^{j}\right)=\left(\theta \pm j,\theta +u\pm j,\ldots ,\theta +\left(T-1\right)u\pm j\right).$$
*Step 3: Construct the LHZ FHS set $B=\left\{b^{p}|0\leq p<kN\right\}$, p=ik+j(0≤j<k,0≤i<N), where for each 0≤p<kN,*
$$b^{p}=b^{ik+j}=I\left(L^{\theta \pm j}\left(a^{i}\right),L^{\theta +u\pm j}\left(a^{i}\right),\ldots ,L^{\theta +(T-1)u\pm j}\left(a^{i}\right)\right)$$

**Theorem 2.**  *The LHZ FHS set B$\left(TL,kN,c,T-1,TH_{m}\left(A\right)\right)$ constructed by Construction 1 is an optimal LHZ FHS set if T satisfies $T\left\lceil \frac{(NL-c)L}{(NL-1)c}\right\rceil =\left\lceil T\frac{(N(L-T)-c)L}{(N(L-T)-1)c}\right\rceil$. By permuting the sequences within a set of shift sequences, the resulting set of sequences is also an optimal LHZ FHS set.*

**Proof of Theorem 2.**  The shift sequence set is represented by a kT matrix,

$$\begin{array}{cc} E= & \left(\begin{array}{cccc} e_{0}^{0} & e_{1}^{0} & \ldots & e_{T-1}^{0}\\ e_{0}^{1} & e_{1}^{1} & \ldots & e_{T-1}^{1}\\ \ldots & \ldots & \ldots & \ldots \\ e_{0}^{k-1} & e_{1}^{k-1} & \ldots & e_{T-1}^{k-1} \end{array}\right)\\ = & \left(\begin{array}{cccc} \theta & \theta +u & \ldots & \theta +(T-1)u\\ \theta \pm 1 & \theta \pm 1+u & \ldots & \theta \pm 1+(T-1)u\\ \cdots & \cdots & \cdots & \cdots \\ \theta \pm (k-1) & \theta \pm (k-1)+u & \ldots & \theta \pm (k-1)+(T-1)u \end{array}\right). \end{array}$$

The $e^{j}$ can be written as the following two cases.

Case 1:

(3) $$\left\{\begin{array}{c} e_{i}^{j}=e_{i}^{j+s}-s,\\ e_{i}^{j}=e_{i+1}^{j+t}-u-t, \end{array}\right.$$

Case 2:

(4) $$\left\{\begin{array}{c} e_{i}^{j}=e_{i}^{j+s}+s,\\ e_{i}^{j}=e_{i+1}^{j+t}-u+t, \end{array}\right.$$

where 0≤i+1≤T−1,0≤j+t,j+s≤k−1. From (3), we have

(5) $$e_{i}^{j}=e_{i}^{j+s}-s=e_{i+1}^{j+t}-u-t.$$

From (5), we have

(6) $$e_{i}^{j+s}=e_{i+1}^{j+t}-u-t+s.$$

We can obtain the relationship between the parameters of the rows and columns in the shift matrix from the above equation.

If any parameter meets the condition s=u+t, then (s−t)T=L. But the maximum value of s−t is k−1. Therefore, the shift sequence $e_{i}^{j}$ are not identical to each other.

It can be learned from (5) that when s=1 and $\tau_{2}=0$, the value

$$\min_{e^{j_{1}},e^{j_{2}}\in E}\left\{\min_{0<t<T}\left\{{\;d}_{t,\tau_{2}}^{e^{j_{1}},e^{j_{2}}}\right\}\right\}=1.$$

Therefore, from the Theorem 2, the LHZ $Z_{h}$ of B is T−1. The same analysis as above for (4), the LHZ $Z_{h}$ of B is T−1.

Concurrently, if the columns of the shift matrix are transformed, different representations of the shift matrix can be obtained. The following shift matrix is one of such representations.

$$\begin{array}{cc} E= & \left(\begin{array}{cccc} e_{v}^{0} & e_{T-1}^{0} & \ldots & e_{1}^{0}\\ e_{v}^{1} & e_{T-1}^{1} & \ldots & e_{1}^{1}\\ \ldots & \ldots & \ldots & \ldots \\ e_{v}^{k-1} & e_{T-1}^{k-1} & \ldots & e_{1}^{k-1} \end{array}\right)\\ = & \left(\begin{array}{cccc} \theta +vu & \theta +(T-1)u & \ldots & \theta +u\\ \theta \pm 1+vu & \theta \pm 1+(T-1)u & \ldots & \theta \pm 1+u\\ \cdots & \cdots & \cdots & \cdots \\ \theta \pm (k-1)+vu & \theta \pm (k-1)+(T-1)u & \ldots & \theta \pm (k-1)+u \end{array}\right), \end{array}$$

where 1<v<T−1. Based on the above proof, it can be shown that each element in a shift matrix is distinct and shift matrices do not collide with each other in rows and columns. Therefore, we have the LHZ $Z_{h}$ of B is T−1.

Moreover, we have

$$H_{m}\left(A\right)=\left\lceil \frac{(NL-c)L}{(NL-1)c}\right\rceil .$$

According to Lemma 2, the MHC $H_{m}\left(B\right)$$\left(TL,kN,c,T-1,H_{m}\left(B\right)\right)$ of FHS set B is

$$\begin{array}{cc} \; & H_{m}\left(B\right)\geq T\frac{(kN(T-1)+kN-c)L}{(kN(T-1)+kN-1)c}=T\frac{(kNT-c)L}{(kNT-1)c}=T\frac{(NT(u-1)-c)L}{(NT(u-1)-1)c}\\ \; & =T\frac{(N(L-T)-c)L}{(N(L-T)-1)c}. \end{array}$$

For $T\left\lceil \frac{(NL-c)L}{(NL-1)c}\right\rceil =\left\lceil T\frac{(N(L-T)-c)L}{(N(L-T)-1)c}\right\rceil$, the MHC of B is the value that satisfies the equal sign of the Peng–Fan–Lee bound. In a word, the LHZ FHS set B is said to be the optimal. □

**Example 1.**  *Select an optimal (16, 3, 7, 2) FHS set $A=\{a^{0},a^{1},a^{2}\}$, where*

$$\begin{array}{cc} \; & a^{0}=\left\{1,0,1,6,2,4,5,6,6,0,6,1,5,3,2,1\right\},\\ \; & a^{1}=\left\{2,3,6,4,2,2,0,2,5,4,1,3,5,5,0,5\right\},\\ \; & a^{2}=\left\{6,3,3,0,3,4,6,5,1,4,4,0,4,3,1,2\right\}. \end{array}$$

*One can obtain the shift sequences $E=\{e^{j}=e_{0}^{j},e_{1}^{j},e_{2}^{j},e_{3}^{j}\},0\leq j<3$, such that $e^{0}=\left\{0,4,8,12\right\},e^{1}=\left\{15,3,7,11\right\},e^{2}=\left\{14,2,6,10\right\}$. It follows that*

$$E=\left(\begin{array}{cccc} e_{0}^{0} & e_{1}^{0} & e_{2}^{0} & e_{3}^{0}\\ e_{0}^{1} & e_{1}^{1} & e_{2}^{1} & e_{3}^{1}\\ e_{0}^{2} & e_{1}^{2} & e_{2}^{2} & e_{3}^{2} \end{array}\right)=\left(\begin{array}{cccc} 0 & 4 & 8 & 12\\ 15 & 3 & 7 & 11\\ 14 & 2 & 6 & 10 \end{array}\right).$$

*Construct the LHZ FHS set $B=\{b^{0},b^{1},b^{2},b^{3},b^{4},b^{5},b^{6},b^{7},b^{8}\}$ by the Construction 1, where*

$$\begin{array}{cc} \; & b^{0}=\left\{1,2,6,5,0,4,0,3,1,\cdots ,1,6,6,1\right\},\;b^{1}=\left\{1,6,6,1,1,2,6,5,0,\cdots ,2,1,5,6\right\},\\ \; & b^{2}=\left\{2,1,5,6,1,6,6,1,1,\cdots ,3,0,4,0\right\},\;b^{3}=\left\{2,2,5,5,3,2,4,5,6,\cdots ,5,4,2,3\right\},\\ \; & b^{4}=\left\{5,4,2,3,2,2,5,5,3,\cdots ,0,6,0,1\right\},\;b^{5}=\left\{0,6,0,1,5,4,2,3,2,\cdots ,5,3,2,4\right\},\\ \; & b^{6}=\left\{6,3,1,4,3,4,4,3,3,\cdots ,2,0,5,0\right\},\;b^{7}=\left\{2,0,5,0,6,3,1,4,3,\cdots ,1,3,6,4\right\},\\ \; & b^{8}=\left\{1,3,6,4,2,0,5,0,6,\cdots ,3,3,4,4\right\}. \end{array}$$

**Figure 1:**
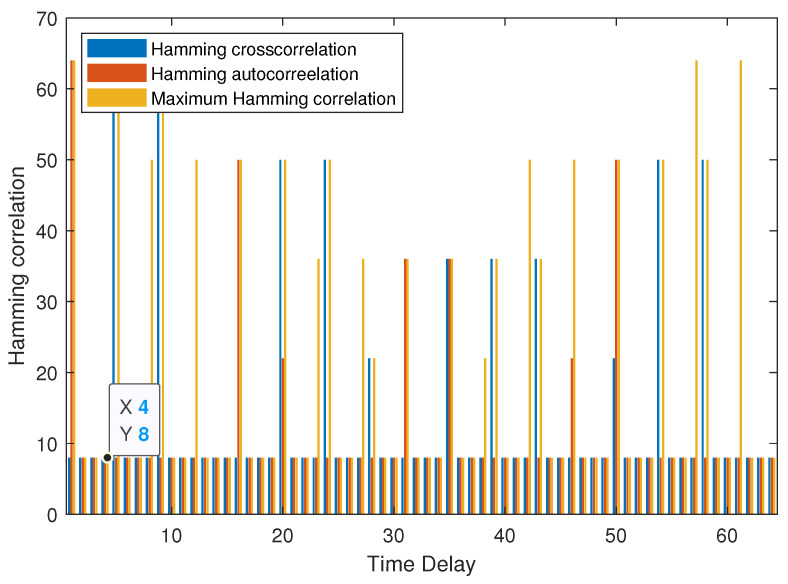
MHC of B in Example 1.

*As shown in Figure 1, the MHC of set B is 8 when the 0<τ≤3. It can be verified that $H_{m}\left(B\right)=8$ for τ≤3, thus B is an optimal (64, 9, 7, 3, 8) LHZ FHS set.*

**Construction 2.**  *Step 1: Select an optimal $\left(L,N,c,H_{m}\left(A\right)\right)$ FHS set A with respect to the Peng–Fan bound.*

$$A=\left\{a^{j}=\left(a_{0}^{j},a_{1}^{j},\ldots ,a_{L-1}^{j}\right)|0\leq j<N\right\}.$$

*Step 2: Select two integers θ,T and a positive integer w, T≥2. Then, generate a shift sequence $E=\{e_{i}|0\leq i<T\}$ as follows,*

$$E=\left(e_{0},e_{1},\ldots ,e_{T-1}\right)=\left(\theta ,\theta +w,\ldots ,\theta +\left(T-1\right)w\right).$$

*Step 3: Construct a new set of FHS $B=\left\{b^{j}=\left\{b^{j}\left(x\right)|0\leq x<TL\right\},0\leq j<L\right\}$,*

$$b^{j}=I\left(L^{\theta }\left(a^{i}\right),L^{\theta +w}\left(a^{i}\right),\ldots ,L^{\theta +(T-1)w}\left(a^{i}\right)\right)$$

**Theorem 3.**  *The LHZ FHS set constructed by Construction 2 is an optimal LHZ FHS set if the parameters meet the following conditions. If $w<\frac{L+1}{2}$, T satisfies $T\left\lceil \frac{(NL-c)L}{(NL-1)c}\right\rceil =\left\lceil \frac{T(N(Tw-1)-c)L}{(N(Tw-1)-1)c}\right\rceil$ then B$\left(TL,N,c,Tw-2,TH_{m}\left(A\right)\right)$ is an optimal LHZ FHS set. If $w>\frac{L+1}{2}$ and T satisfies $T\left\lceil \frac{(NL-c)L}{(NL-1)c}\right\rceil =\left\lceil \frac{(NT(L-w)+N-c)TL}{(NT(L-w)+N-1)c}\right\rceil$ then B$\left(TL,N,c,T\left(L-w\right),TH_{m}\left(A\right)\right)$ is an optimal LHZ FHS set.*

**Proof of Theorem 3.**  We have

$$d_{t,\tau_{2}}^{\left(e,e\right)}=\left\{\begin{array}{c} e_{t}-e_{t+\tau_{2}},0\leq t\leq T-1+\tau_{2}\\ e_{t}-e_{t+\tau_{2}-T}-1,T-\tau_{2}\leq t\leq T-1 \end{array}\right..$$

From the parameters of the shift sequence set in Construction 2, we have

$$\begin{array}{c} d_{t,\tau_{2}}^{\left(e,e\right)}=\left\{\begin{array}{c} -w\tau_{2},0\leq t\leq T-1+\tau_{2}\\ w\left(T-\tau_{2}\right)-1,T-\tau_{2}\leq t\leq T-1 \end{array}\right.. \end{array}$$

Therefore, if $w<\frac{L+1}{2}$ and $\tau_{2}=T-1$, the minimum value of $d_{t,\tau_{2}}^{\left(e,e\right)}$ is w−1. Then,

$$Z_{h}=T\left(w-1\right)+\tau_{2}-1=Tw-2.$$

If $w>\frac{L+1}{2}$ and $\tau_{2}=1$, the minimum value of $d_{t,\tau_{2}}^{\left(e,e\right)}$ is L−w. Then,

$$Z_{h}=T\left(L-w\right).$$

Furthermore, we have

$$H_{m}\left(A\right)=\frac{(NL-c)L}{(NL-1)c}.$$

According to Lemma 2, the MHC $H_{m}\left(B\right)$ of FHS B$\left(TL,N,c,H_{m}\left(B\right)\right)$ is

Case 1: when $w<\frac{L+1}{2}$,

$$\begin{array}{cc} \; & H_{m}\left(B\right)\geq \frac{\left(NZ+N-c\right)L}{\left(NZ+N-1\right)c}=\frac{(N(Tw-2)+N-c)TL}{(N(Tw-2)+N-1)c}=\frac{(N(Tw-1)-c)TL}{(N(Tw-1)-1)c}. \end{array}$$

For

$$T\left\lceil \frac{(NL-c)L}{(NL-1)c}\right\rceil =\left\lceil \frac{T(N(Tw-1)-c)L}{(N(Tw-1)-1)c}\right\rceil ,$$

the MHC of B is the value that satisfies the equal sign of the Peng–Fan–Lee bound.

Case 2: when $w>\frac{L+1}{2}$,

$$\begin{array}{cc} \; & H_{m}\left(B\right)\geq \frac{\left(NZ+N-c\right)TL}{\left(NZ+N-1\right)c}=\frac{(NT(L-w)+N-c)TL}{(NT(L-w)+N-1)c} \end{array}$$

For

$$T\left\lceil \frac{(NL-c)L}{(NL-1)c}\right\rceil =\left\lceil T\frac{(NT(L-w)+N-c)L}{(NT(L-w)+N-1)c}\right\rceil ,$$

the MHC of B is the value that satisfies the equal sign of the Peng–Fan–Lee bound.

Therefore, the LHZ FHS set B is said to be the optimal. □

**Example 2.**  *Select an optimal (16, 3, 7, 2) FHS set $A=\{a^{0},a^{1},a^{2}\}$, where*

$$\begin{array}{cc} \; & a^{0}=\left\{1,0,1,6,2,4,5,6,6,0,6,1,5,3,2,1\right\},\\ \; & a^{1}=\left\{2,3,6,4,2,2,0,2,5,4,1,3,5,5,0,5\right\},\\ \; & a^{2}=\left\{6,3,3,0,3,4,6,5,1,4,4,0,4,3,1,2\right\}. \end{array}$$

*We set the parameters θ=1,w=2,T=5. Then, the shift sequence is E={1,3,5,7,9}.*

*Construct the LHZ FHS set $B=\{b^{0},b^{1},b^{2}\}$ where*

$$\begin{array}{cc} \; & b^{0}=\left\{0,6,4,6,0,1,2,5,6,6,6,4,6,0,\cdots ,6,1,1,2,5,6\right\}\\ \; & b^{1}=\left\{3,4,2,2,4,6,2,0,5,1,4,2,2,4,\cdots ,2,2,6,2,0,5\right\}\\ \; & b^{2}=\left\{3,0,4,5,4,3,3,6,1,4,0,4,5,4,\cdots ,5,6,3,3,6,1\right\} \end{array}$$

**Figure 2:**
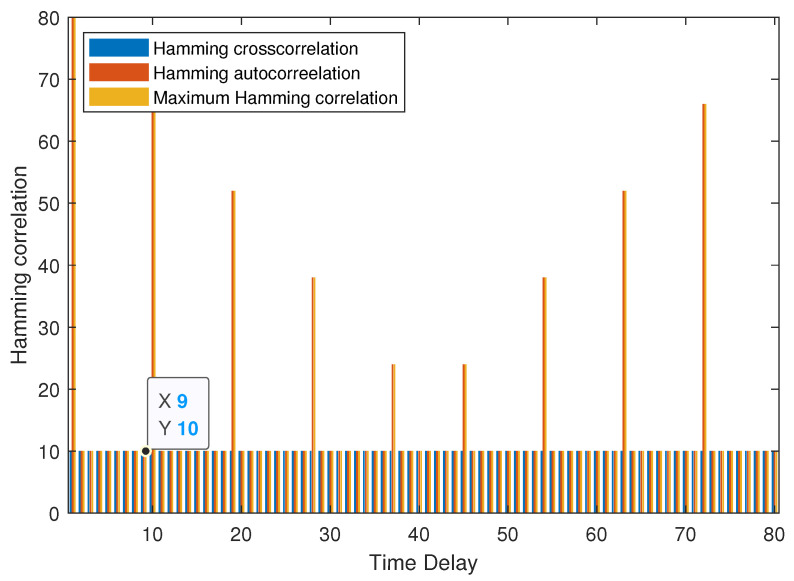
MHC of B in Example 2.

*As shown in Figure 2, the MHC of set B is 10 when the τ≤8. It can be verified that $\left\lceil \frac{\left(NZ+N-c\right)L}{\left(NZ+N-1\right)c}\right\rceil =10$, then B is an optimal (80, 3, 7, 8, 10) LHZ FHS set.*

**Example 3.**  *Select an optimal (16, 7, 3, 2) FHS set $A=\{a^{0},a^{1},a^{2}\}$, such that*

$$\begin{array}{cc} \; & a^{0}=\left\{1,0,1,6,2,4,5,6,6,0,6,1,5,3,2,1\right\},\\ \; & a^{1}=\left\{2,3,6,4,2,2,0,2,5,4,1,3,5,5,0,5\right\},\\ \; & a^{2}=\left\{6,3,3,0,3,4,6,5,1,4,4,0,4,3,1,2\right\}. \end{array}$$

*We set the parameters θ=1,w=14,T=4, the shift sequence is E={1,15,13,11}.*

*Construct the LHZ FHS set $B=\{b^{0},b^{1},b^{2}\}$ where*

$$\begin{array}{cc} \; & b^{0}=\left\{0,1,3,1,1,1,2,5,6,0,1,3,2,1,1,\cdots ,1,0,1,2,5,6\right\},\\ \; & b^{1}=\left\{3,5,5,3,6,2,0,5,4,3,5,5,2,6,2,\cdots ,3,4,2,0,5,1\right\},\\ \; & b^{2}=\left\{3,2,3,0,3,6,1,4,0,3,2,3,3,3,6,\cdots ,0,4,6,1,4,4\right\}. \end{array}$$

**Figure 3:**
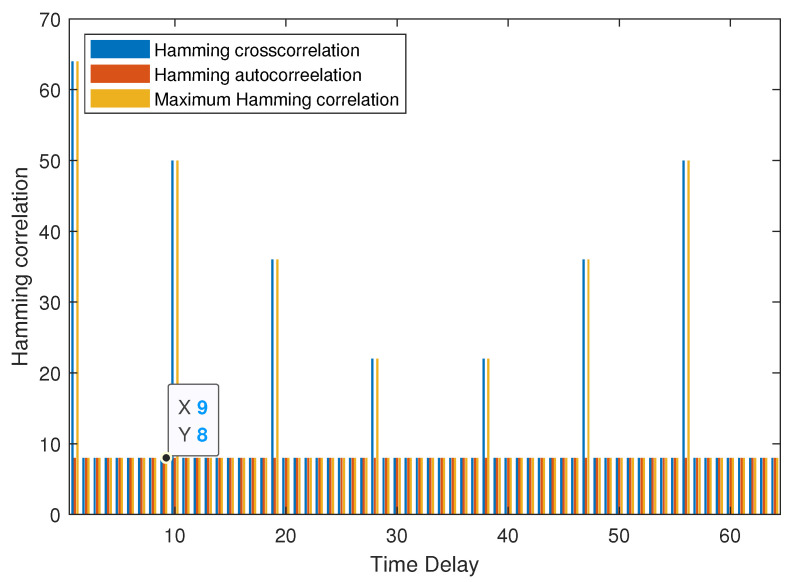
MHC of B in Example 3.

*As shown in Figure 3, the MHC of set B is 8 when the time delay τ≤8. It can be verified that $\left\lceil \frac{\left(NZ+N-c\right)L}{\left(NZ+N-1\right)c}\right\rceil =8$, then B is an optimal (64, 3, 7, 8, 8) LHZ FHS set.*

**Construction 3.**  *Step 1: Select an optimal $\left(L,N,c,H_{m}\left(A\right)\right)$ FHS set A that satisfies the Peng–Fan bound, $A=\left\{a^{i}=\left(a_{0}^{i},a_{i}^{i},\ldots ,a_{L-1}^{i}\right),0\leq i<N\right\}$.*

*Step 2: Let T, u, k be three positive integers. w, θ are two integers, T>2, w>1 and w=θ+uT. The shift sequence is $E=\left\{e_{i}^{j}|0\leq i<T,0\leq j<k\right\}$. We have*
$$\begin{array}{c} e^{j}=\left(e_{0}^{j},e_{1}^{j},\ldots ,e_{T-1}^{j}\right)=\left(\theta +jw,\theta +u+jw,\ldots ,\theta +\left(T-1\right)u+jw\right). \end{array}$$
*Step 3: Construct LHZ FHS set $B=\left\{b^{p}|0\leq p<kN\right\}$, p=ik+j(0≤i<N,0≤j<k), where for each 0≤p<kN,*
$$b^{p}=I\left(L^{\theta +jw}\left(a^{i}\right),L^{\theta +u+jw}\left(a^{i}\right),\ldots ,L^{\theta +(T-1)u+jw}\left(a^{i}\right)\right).$$

**Theorem 4.**  *The LHZ FHS set B constructed by Construction 3 is an optimal B(TL,kN,c,2T−2,$TH_{m}\left(A\right))$ LHZ FHS set if parameter meets k(2T−1)≤L, u(kT−1)=L−1 and $T\left\lceil \frac{(NL-c)L}{(NL-1)c}\right\rceil =\left\lceil T\frac{(kN(2T-1)-c)L}{(kN(2T-1)-1)c}\right\rceil$.*

**Proof of Theorem 4.**  The shift sequence set is represented by a kT matrix,

$$\begin{array}{cc} E= & \left(\begin{array}{cccc} e_{0}^{0} & e_{1}^{0} & \ldots & e_{T-1}^{0}\\ e_{0}^{1} & e_{1}^{1} & \ldots & e_{T-1}^{1}\\ \ldots & \ldots & \ldots & \ldots \\ e_{0}^{k-1} & e_{1}^{k-1} & \ldots & e_{T-1}^{k-1} \end{array}\right)\\ = & \left(\begin{array}{cccc} \theta & \theta +u & \ldots & \theta +(T-1)u\;\\ \theta +w & \theta +u+w & \ldots & \theta +(T-1)u+w\\ \ldots & \ldots & \ldots & \ldots \\ \theta +(k-1)w & \theta +u+(k-1)w\; & \ldots \; & \theta +(T-1)u+(k-1)w \end{array}\right) \end{array}$$

The $e^{j}$ can be written as

(7) $$\begin{array}{c} \left\{\begin{array}{c} e_{i}^{j}=e_{i+s}^{j-s}-s\left(u-w\right),\\ e_{i}^{j}=e_{i}^{j\pm 1}\pm w, \end{array}0<i+s,j-s<T\right. \end{array}$$

From (7), we have

(8) $$e_{i}^{j}=e_{i+s}^{j-s}-s\left(u-w\right)=e_{i}^{j\pm 1}\pm w.$$

From (8), we have

(9) $$e_{i+s}^{j-s}=e_{i}^{j\pm 1}\pm w+s\left(u-w\right).$$

For u(kT−1)=L−1, then θ−(θ+(T−1)u+(k−1)w) (mod *L*) = 1. From (8) and (9), ±w (mod *L*) ≥ 1, sw−su (mod *L*) ≥1 for T>2 and w=θ+uT. Thus,

$$\min_{e^{j_{1}},e^{j_{2}}\in E}\left\{\min_{0<i<T}\left\{\;d_{t,\tau_{2}}^{e^{j_{1}},e^{j_{2}}}\right\}\right\}=1.$$

At this time, the delay time $\tau_{2}$ is T−1, then

$$Z_{h}=\left\{Td_{t,\tau }^{\left(e^{j_{1}},e^{j_{2}}\right)}+\tau_{2}\right\}-1=2T-2.$$

Besides, we have

$$H_{m}\left(A\right)=\frac{(NL-c)L}{(NL-1)c},$$

According to Lemma 2, the MHC $H_{m}\left(B\right)$$\left(TL,kN,c,H_{m}\left(B\right),2T-2\right)$ of LHZ FHS set B is

$$\begin{array}{cc} \; & H_{m}\left(B\right)\geq \frac{(kN(2T-2)+kN-c)TL}{(kN(2T-2)+kN-1)c}=T\frac{(kN(2T-1)-c)L}{(kN(2T-1)-1)c}. \end{array}$$

For

$$T\left\lceil \frac{(NL-c)L}{(NL-1)c}\right\rceil =\left\lceil T\frac{(kN(2T-1)-c)L}{(kN(2T-1)-1)c}\right\rceil ,$$

the MHC of B is the value that satisfies the equal sign of Peng-Fan-Lee bound. All in all, the LHZ FHS set B is said to be the optimal. □

**Example 4.**  *Select an optimal (16, 3, 7, 2) FHS set $A=\{a^{0},a^{1},a^{2}\}$, where*

$$\begin{array}{cc} \; & a^{0}=\left\{1,0,1,6,2,4,5,6,6,0,6,1,5,3,2,1\right\},\\ \; & a^{1}=\left\{2,3,6,4,2,2,0,2,5,4,1,3,5,5,0,5\right\},\\ \; & a^{2}=\left\{6,3,3,0,3,4,6,5,1,4,4,0,4,3,1,2\right\}. \end{array}$$

*We set the parameters θ=0,T=3,k=2,w=9,u=3, the shift sequences $e^{0}=\left\{0,3,6\right\}$, $e^{1}=\left\{9,12,15\right\}$. It follows that*

$$E=\left(\begin{array}{ccc} e_{0}^{0} & e_{1}^{0} & e_{2}^{0}\\ e_{0}^{1} & e_{1}^{1} & e_{2}^{1} \end{array}\right)=\left(\begin{array}{ccc} 0 & 3 & 6\\ 9 & 12 & 15 \end{array}\right).$$

*We construct the LHZ FHS set $B=\{b^{0},b^{1},b^{2},b^{3},b^{4},b^{5}\}$ where*

$$\begin{array}{cc} \; & b^{0}=\left\{1,6,5,0,2,6,1,4,6,6,5,0,\cdots ,2,0,2,1,1,4\right\},\\ \; & b^{1}=\left\{0,5,1,6,3,1,1,2,0,5,1,1,\cdots ,6,6,3,6,1,2\right\},\\ \; & b^{2}=\left\{2,4,0,3,2,2,6,2,5,4,0,4,\cdots ,0,3,2,5,6,2\right\},\\ \; & b^{3}=\left\{4,5,5,1,5,2,3,0,3,5,5,6,\cdots ,2,1,5,5,3,0\right\},\\ \; & b^{4}=\left\{6,0,6,3,3,5,3,4,1,0,6,4,\cdots ,1,3,3,2,3,4\right\},\\ \; & b^{5}=\left\{4,4,2,4,3,6,0,1,3,4,2,3,\cdots ,5,4,3,1,0,1\right\}. \end{array}$$

**Figure 4:**
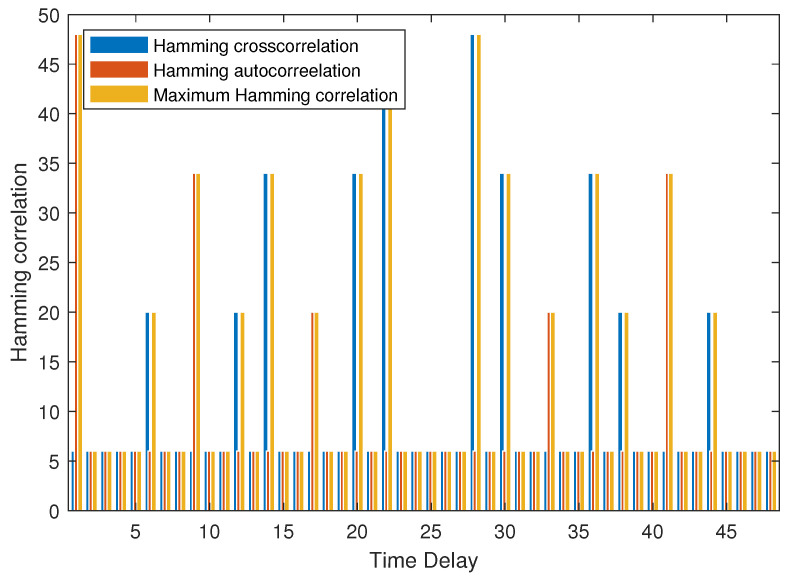
MHC of B in Example 4.

*As shown in Figure 4, the MHC of set B is 6 when the τ≤4. It can be verified that $\left\lceil \frac{\left(NZ+N-c\right)L}{\left(NZ+N-1\right)c}\right\rceil =6$, then B is an optimal (48, 6, 7, 4, 6) LHZ FHS set.*

## 5. Conclusions

In this paper, we propose three new methods for the construction of the optimal LHZ FHS set and prove some sufficient conditions that they need to satisfy. As a comparison, we list the parameters of the existing optimal LHZ FHS sets and the optimal LHZ FHS sets constructed in this paper in Table 1. As a result, our constructed sequences are more flexible and can be used to eliminate MI in QS FHMA systems. Future work can explore the application of FHSs in more scenarios, such as image processing, data encryption, mobile communication, security and privacy. Overall, exploring the application of FHSs in various scenarios can lead to new innovations and improvements in different areas of wireless communication.

## Figures and Tables

**Table 1:** Comparison of parameters for LHZ FHS sets with optimal Hamming correlation.

Parameters $\left(L,N,c,Z_{h},H_{m}\left(Q\right)\right)$	Constraints	Ref.
$(s\left(q^{n}-1\right),M,q,w-1,$ $s(q^{n-1}-1))$	$q^{n}-1=wm,gcd\left(s,q^{n}-1\right)=1.$	[6]
$\left(s\left(p^{n}-1\right),e,e+1,w-1,sf\right)$	$gcd\left(s,p^{n}-1\right)=1,w=\frac{p^{n}-1}{m},m|\left(p^{n}-1\right),$ 1≤m<f, e+1>sf, $sfe^{2}m<\left(fe^{2}-m\right)\left(e+1-sf\right).$	[13]
(sN,mM,v,w−1,sλ)	$m=\lceil \frac{N}{w}\rceil$, gcd(s,N)=1,s=aw+1, a≥1, s<mN.	[9]
(MN,m,v,wM−1,Mλ)	$m=\lceil \frac{N}{w}\rceil$.	[8]
(MN,m,v,M−2,Mλ)	$m=\lceil \frac{N}{w}\rceil$, w>2M.	[16]
$\left(TL,kN,c,T-1,TH_{m}\left(A\right)\right)$	uT=L, k=u−1, $T\left\lceil \frac{(NL-c)L}{(NL-1)c}\right\rceil =\left\lceil T\frac{(N(L-T)-c)L}{(N(L-T)-1)c}\right\rceil$	Construction 1
$\left(TL,N,c,Tw-2,TH_{m}\left(A\right)\right)$	$w<\frac{L+1}{2}$, $T\left\lceil \frac{(NL-c)L}{(NL-1)c}\right\rceil =\left\lceil \frac{T(N(Tw-1)-c)L}{(N(Tw-1)-1)c}\right\rceil$	Construction 2
$\left(TL,N,c,T\left(L-w\right),TH_{m}\left(A\right)\right)$	$w>\frac{L+1}{2}$, $T\left\lceil \frac{(NL-c)L}{(NL-1)c}\right\rceil =\left\lceil \frac{(NT(L-w)+N-c)TL}{(NT(L-w)+N-1)c}\right\rceil$	Construction 2
$\left(TL,kN,c,2T-2,TH_{m}\left(A\right)\right)$	k(2T−1)≤L, u(kT−1)=L−1, $T\left\lceil \frac{(NL-c)L}{(NL-1)c}\right\rceil =\left\lceil T\frac{(kN(2T-1)-c)L}{(kN(2T-1)-1)c}\right\rceil$	Construction 3

## Data Availability

Not applicable.

## Abbreviations

The following abbreviations are used in this manuscript:

FHS	frequency-hopping sequence
HC	Hamming correlation
LHZ	low-hit-zone
MHAC	maximum Hamming autocorrelation
MHC	maximum Hamming correlation
MHCC	maximum Hamming crosscorrelation
MI	mutual interference
QS-FHMA	quasi-synchronous frequency-hopping multiple access
